# Availability of the core components of the World Health Organization infection prevention and control strategies in health facilities in Southwestern Uganda: Implications for control of COVID-19

**DOI:** 10.1016/j.infpip.2022.100206

**Published:** 2022-02-10

**Authors:** Richard Ssekitoleko, Emmanuel Seremba, Florence Waiswa, Doreen Nabawanuka, Paul Muyinda, Solome Okware, Bongomin Bodo, Yonas Tegegn Woldemariam, Christopher C. Moore

**Affiliations:** a World Health Organization, Kampala, Uganda; b College of Health Sciences, Makerere University, Kampala, Uganda; c Kiruddu National Hospital, Kampala, Uganda; d College of Education and External Studies, Makerere University, Kampala, Uganda; e Division of Infectious Diseases and International Health, University of Virginia, USA; f Department of Medicine, Mbarara University of Science and Technology, Uganda

**Keywords:** Infection prevention and control, COVID-19, Africa, Uganda

## Abstract

**Background:**

Infection prevention and control (IPC) practices are required to prevent nosocomial infection by severe acute respiratory syndrome coronavirus 2. In low- and middle-income countries, where resources are often limited, IPC practices are infrequently assessed.

**Aim:**

To assess the availability of the core components of World Health Organization (WHO) IPC practices at health facilities in Southwestern Uganda.

**Methods:**

We assessed the availability of WHO IPC core components using a modified WHO IPC Assessment tool. We determined differences between government versus private ownership and by type of health facility.

**Findings:**

We assessed 111 of 224 (50%) health facilities in four districts. The most frequently achieved core component of IPC strategies was environmental cleanliness with 75 of 111 (68%) facilities scoring >85%. The most infrequently achieved core component of IPC strategies was personal protective equipment (PPE) with only one of seven (14%) hospitals and no other facilities scoring >85%. Of the 20 hospital or health center IV facilities, five (25%) received an overall score of >85% compared to only one of 91 (1%) health center II or III facilities (odds ratio [OR] 30.0 [95% CI: 3.27–274.99], p=0.003). Of the 73 government facilities, two (3%) received an overall score of >85% compared to five of 38 (13%) private facilities (OR 0.24 [95% CI: 0.04–1.37], p=0.11).

**Conclusion:**

Few facilities in four districts in Southwestern Uganda achieved >85% availability of WHO IPC core components. Provision of PPE in these facilities should be prioritized.

## Introduction

From the time of its discovery in 2019 until November, 2021, there have been over 255 million confirmed cases of severe acute respiratory syndrome coronavirus 2 (SARS-CoV-2) infection and over 5.1 million associated global deaths [1]. During the same time period, there have been over 7.1 million confirmed cases of Coronavirus Disease-2019 (COVID-19) and over 170,000 associated deaths in Africa. Given limited access to testing facilities and case reporting in Africa, the number of cases of COVID-19 and attributable deaths are likely underestimated [2]. Furthermore, by November 2021, only 6% of eligible persons in Africa had been vaccinated. Although access to vaccines is improving, the vaccinated population remains dismally low, so the number of COVID-19 cases and deaths is likely to continue unabated in Africa for the foreseeable future [3].

While most patients with COVID-19 have mild symptoms, many require hospitalization and critical care [4]. In Uganda, intensive care units (ICUs) that could be used to cohort and treat critically ill COVID-19 patients are not available in most health facilities [5]. Accordingly, before patients with COVID-19 in Uganda are identified and transferred to dedicated COVID-19 treatment facilities, they may initially present to district health facilities or hospital general wards where infection control and prevention can be challenging.

In order to prevent nosocomial transmission of pathogens including SARS-CoV-2, the World Health Organization (WHO) has provided recommendations for Infection Prevention and Control (IPC) strategies. The core components of these strategies include 1) ensuring triage, early recognition, and source control (isolating or cohorting patients with suspected COVID-19), 2) applying standard IPC precautions for all patients, 3) implementing empiric additional precautions (droplet and contact and, whenever applicable, airborne precautions) for suspected cases of COVID-19, 4) implementing administrative controls, and 5) using environmental and engineering controls [6]. The WHO has also developed the Infection Prevention and Control Assessment Framework (IPCAF) tool to measure the level of IPC implementation, which was recently validated in 181 hospitals in 46 countries [7]. In addition, the WHO has provided interim guidance for IPC assessments in health care facilities in the setting of confirmed or suspected cases of hemorrhagic fever as well as during the COVID-19 pandemic [6,8].

Following the declaration of the Ebola Viral Disease (EVD) outbreak in the Democratic Republic of the Congo (DRC) in August 2018, the WHO identified Uganda as at high risk for an EVD outbreak. Based on this assessment, interventions to improve IPC practices were put in place in the high-risk districts in Uganda bordering the DRC, including the Southwestern region districts of Kabale, Kanungu, Kisoro, Rubirizi, and Rukungiri [9]. In this study, using a modified WHO IPC assessment tool, we aimed to assess the availability of the WHO core components of IPC strategies in the health facilities in these districts.

## Methodology

### Study design and health facility selection

The study adopted a mixed methods research design involving a health facility-based survey and interview of key personnel at selected health facilities. Uganda Ministry of Health district health officers provided a complete list of health facilities in each district to the WHO field assessors. We conducted a convenience sampling of government and private health centers II, III and IV, and general hospitals in the four districts prioritizing the inclusion of health center IVs and hospitals.

### Survey population

The survey population included all government and private health facilities in four Southwestern districts of Uganda including Kabale, which borders Rwanda; and Kanungu, Rubirizi and Rukungiri, which border the DRC. In aggregate, the districts are inhabited by 1,003,900 people (Kabale, 248,700; Kanungu, 277,300; Rubirizi, 144,100; and Rukungiri, 333,800) and have 224 health facilities comprised of 64 in Kabale, 54 in Kanungu, 16 in Rubirizi, and 90 in Rukungiri [10].

The health care infrastructure in Uganda includes government facilities, private facilities which may be Private Not for Profit (PNFP) or Private for Profit (PFP), as well as traditional and complementary medicine facilities. National referral hospitals provide the highest level of service through comprehensive medical and surgical care. General hospitals provide a range of services which may be preventive, promotive, or curative, maternity inpatient care, surgery, blood transfusion, laboratory and radiographic imaging. They also provide in-service training, consultation, and operational research. Health center IV facilities employ nurses, doctors, and laboratory staff, and provide county level care including medical and surgical treatments. Health center III facilities employ nurses, clinical officers, and laboratory staff; and provide basic preventative, promotive, and therapeutic care, including in some cases inpatient admission at the sub-county level. Health center II facilities employ nursing staff and perform outpatient and community outreach activities at the parish level [11].

### Data collection, procedures, and definitions

The WHO IPCAF tool can be used to conduct evaluations of IPC practices and allow health facilities to monitor and facilitate the advancement of IPC practices over time. The questions in the WHO IPCAF tool are structured to mirror the WHO core components of IPC [12]. Based on the responses collected by the tool, a facility may be given one of four outcomes: inadequate, basic, intermediate, or advanced. Since not all components of IPC practices are available at all health facilities in Uganda, the IPCAF tool was modified by the Uganda WHO country office IPC committee according to interim WHO guidance in the setting of suspected or confirmed cases of hemorrhagic fever to function as a checklist tailored to all health facilities for the assessment of available IPC activities and programmes during the preparedness, response, and recovery phases of an EVD epidemic in Uganda [8].

**Figure 1:**
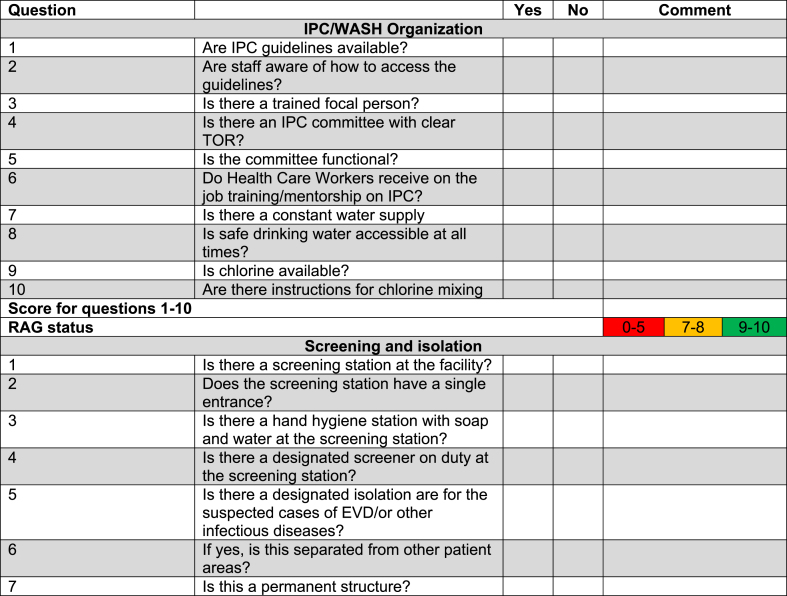

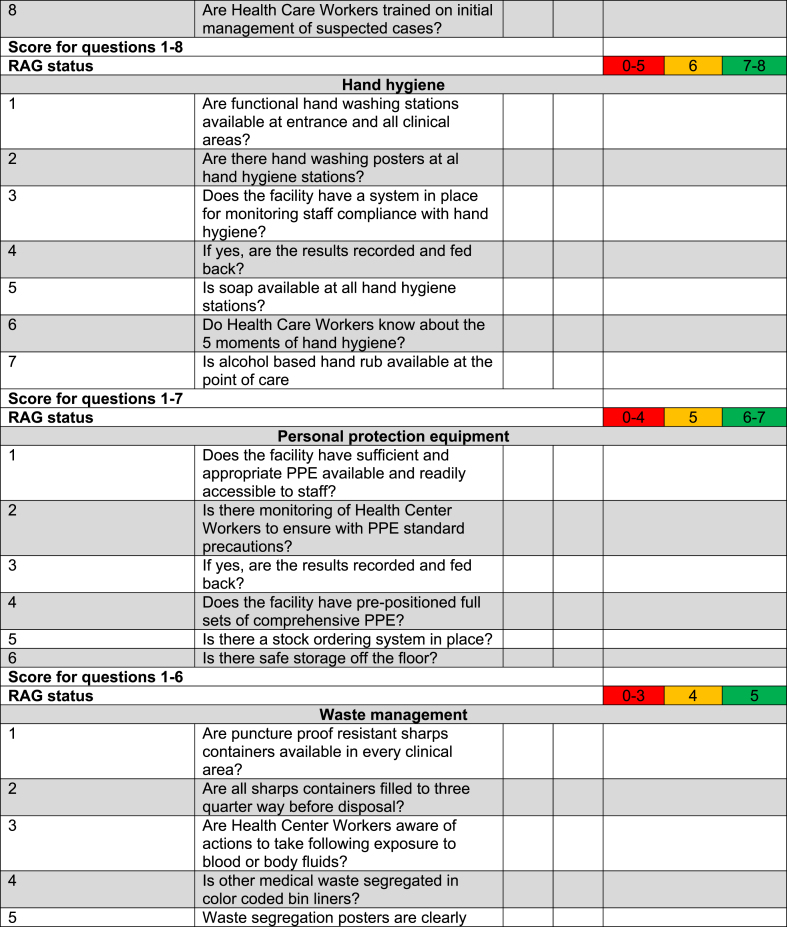

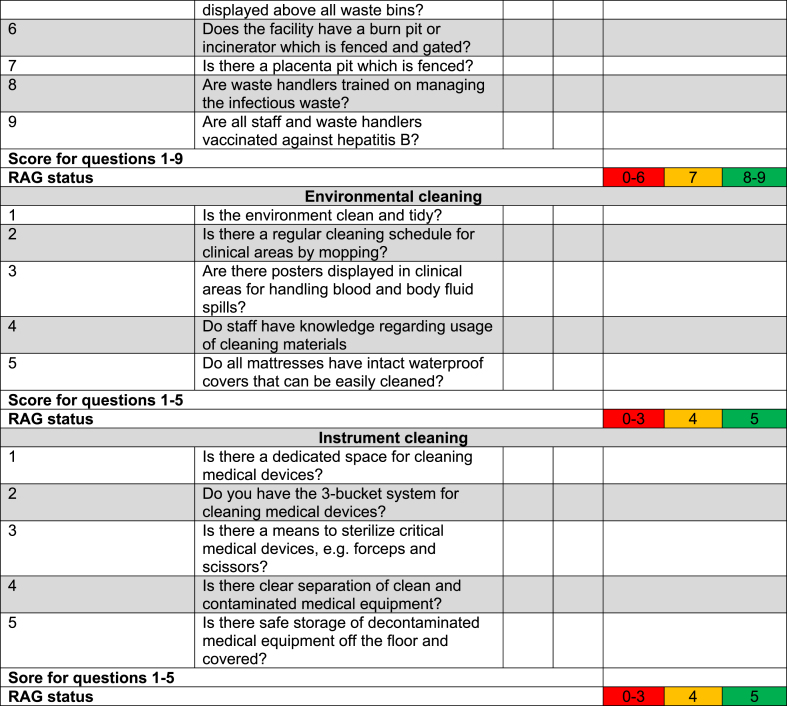
Modified WHO Infection Prevention and Control Assessment tool used to assess for core components of the World Health Organization Infection Prevention and Control strategies in health facilities in Southwestern Uganda.

The modified WHO IPC assessment tool was used for IPC assessments during the EVD preparedness phase and, as per WHO interim guidance for IPC during the COVID-19 pandemic, is now in use for IPC assessments of health facilities as part of the COVID-19 pandemic response in Uganda [6]. The modified WHO IPC assessment tool includes seven key sections: 1) infection prevention and control/water sanitation and hygiene (IPC/WASH organization), 2) screening and isolation, 3) hand hygiene, 4) personal protective equipment (PPE), 6) waste management, and 7) environmental cleaning and instrument reprocessing (Figure 1). Core components, which were not expected to be available at lower level health facilities, including health facility acquired infection surveillance, use of multimodal strategies for implementation of IPC interventions, as well as assessment of workload, staffing, and bed occupancy were not included in the modified WHO IPC assessment tool.

The modified WHO IPC assessment tool assigns outcomes by a Red, Amber, or Green category system. To determine the appropriate category, aggregate point scores for each subsection were recorded and reported as a percentage of the subtotal. The total score was the aggregate point score and percentage for all subsections out of a maximum of 100 points. For ease of interpretation of results, similar to the WHO IPCAF performance categories, the Red category corresponded to a score of up to 70% (inadequate), the Amber category corresponded to a score of 70–85% (intermediate), and the Green category corresponded to scores above 85% (advanced). Following this development, the modified WHO IPC assessment tool was then transferred to an Open Data Kit mobile telephone application, which was downloaded to the assessors' telephones for use. WHO field consultants visited outpatient and inpatient departments, maternity wards and labor suites, operating theatres, and waste disposal sites to establish if each component was in place using the modified WHO IPC assessment tool. The presence of the core components in the different areas was noted by direct observation or by conducting inquiries with the available staff at the points of assessment.

### Ethical considerations

IPC assessments were required by the Uganda Ministry of Health as part of the ongoing EVD preparedness and COVID-19 emergency response activities. Before commencing the assessment activities, the WHO field team members attended district task force meetings in the study districts. The district task forces consisted of multi-sectoral teams of people from different fields and was set-up in each district to manage EVD preparedness and COVID-19 response activities. During the meetings, the task force members were informed about the planned assessments in all the facilities and provided consent. District health officers also reviewed and approved the data collection tools, and provided verbal consent to allow assessors to visit health facilities in each district. At each facility, unit in-charge personnel provided verbal consent to assessors to allow them to proceed to different departments for the assessment. Staff working in the departments provided verbal consent before being interviewed by assessors about the IPC components available at the facility.

The epidemic preparedness and response activities in the region were supported by the WHO, United Nations Children's Fund (UNICEF), Makerere University Infectious Diseases Institute and USAID Regional Health Integration to Enhance Services in Southwest Uganda Project. Specifically, the WHO assisted with Case Management, IPC capacity building, surveillance and case detection. The WHO also provided technical and logistical support to the health facilities working in concert with the Uganda Ministry of Health and the District Directorate of Health Services.

### Data analysis

We entered data into Microsoft Excel (version 2016, Redmond, Washington) then processed and analyzed them in Stata (version 15, College Station, Texas). We expressed the total number of health facilities assessed in each district as a count and percentage of health facilities. We summed the subtotals for the core components and expressed them as percentages in order to assign them to the appropriate Red, Amber, or Green color scale category. We recorded frequencies and percentages for health facilities and stratified them according to ownership, district, and type of health facility. We categorized ownership as either government or private. Private facilities were either PFP or PNFP. Due to the low numbers within the different types of health facilities, we analyzed hospital and health center IV facilities together as one group, and health center II and III facilities together as a second group. We determined the association of ownership type and health facility groups to the overall outcome based on the Red and Green scale using logistic regression. We considered confidence intervals (CIs) that did not include the null to be statistically significant.

## Results

From January 2020 to July 2020, we assessed 111 of the 224 (50%) health facilities in the four Districts including all seven general hospitals, 13 health center IVs, 43 health center IIIs, and 48 health center IIs. Of the 111 assessed facilities, 13 were in Kabale, 33 in Kanungu, 15 in Rubirizi, and 50 in Rukungiri district (Table 1). The most frequently achieved core component of IPC strategies was environmental cleanliness with six of the seven (86%) general hospitals, 11 of 13 (85%) health center IV, 29 of 43 (67%) health center III and 29 of 48 (60%) health center II facilities scoring >85%. The most infrequently achieved core component of IPC strategies was personal protective equipment (PPE) with only one of seven (14%) hospitals and no other facilities scoring >85%. Overall, three (43%) general hospitals, two (15%) health center IV facilities, one (2%) health center III facility, and no health center II received an overall score >85% for all of the assessed WHO IPC core components (Table 2).

**Table I:** Health facilities in districts in Southwestern Uganda assessed for core components of the World Health Organization Infection Prevention and Control strategies as a proportion of the total number of facilities in the district

District	Health facilities, N	Health facilities assessed, N (%)
**Kabale**		
Government	47	10 (21)
Private not for profit	9	2 (22)
Private for profit	8	1 (12)
**Subtotal**	**64**	**13 (20)**
**Kanungu**		
Government	28	21 (75)
Private not for profit	20	9 (45)
Private for profit	6	3 (50)
**Subtotal**	**54**	**33 (61)**
**Rubirizi**		
Government	11	10 (91)
Private not for profit	3	3 (100)
Private for profit	2	2 (100)
**Subtotal**	**16**	**15 (94)**
**Rukungiri**		
Government	55	32 (58)
Private not for profit	30	14 (47)
Private for profit	5	4 (80)
**Subtotal**	**90**	**50 (56)**
**Total**	**224**	**111 (50)**

**Table II:** Modified WHO Infection Prevention and Control Assessment tool scores of health facilities in Southwestern Uganda based on the Red-Amber-Green status score assessment result for each core component of the World Health Organization Infection Prevention and Control strategies

Core component	Facilities assessed, N	Number of health facilities scoring Red (<70%), N (%)	Number of health facilities scoring Amber (70–85%), N (%)	Number of facilities scoring Green (>85%), N (%)
**IPC/WASH organization**				
Hospital	7	--	--	7 (100)
Health center IV	13	--	1 (8)	12 (92)
Health center III	43	7 (16)	13 (30)	23 (53)
Health center II	48	16 (33)	24 (50)	8 (17)
Subtotal	111	23 (21)	38 (34)	50 (45)
**Screening and isolation**				
Hospital	7	--	2 (29)	5 (71)
Health center IV	13	--	8 (62)	5 (38)
Health center III	43	7 (16)	30 (70)	6 (14)
Health center II	48	5 (10)	38 (79)	5 (10)
Subtotal	111	12 (11)	78 (70)	21 (19)
**Hand hygiene**				
Hospital	7	--	5 (71)	2 (29)
Health center IV	13	4 (31)	9 (69)	--
Health center III	43	11 (26)	31 (72)	1 (2)
Health center II	48	10 (21)	37 (77)	1 (2)
Subtotal	111	25 (22)	82 (74)	4 (4)
**Personal protective equipment**				
Hospital	7	1 (14)	5 (71)	1 (14)
Health center IV	13	4 (31)	9 (69)	--
Health center III	43	19 (44)	24 (56)	--
Health center II	48	31 (65)	17 (35)	--
Subtotal	111	55 (49)	55 (49)	1 (1)
**Waste management**				
Hospital	7	--	6 (86)	1 (14)
Health center IV	13	3 (23)	10 (77)	--
Health center III	43	9 (21)	30 (70)	4 (9)
Health center II	48	19 (40)	29 (60)	--
Subtotal	111	31 (28)	75 (68)	5 (5)
**Environmental cleaning**				
Hospital	7	1 (14)	--	6 (86)
Health center IV	13	--	2 (15)	11 (85)
Health center III	43	7 (16)	7 (16)	29 (67)
Health center II	48	7 (15)	12 (25)	29 (60)
Subtotal	111	15 (14)	21 (19)	75 (68)
**Instrument processing**				
Hospital	7	--	1 (14)	6 (86)
Health center IV	13	--	--	13 (100)
Health center III	43	12 (28)	11 (26)	20 (47)
Health center II	48	44 (92)	4 (8)	--
Subtotal	111	56 (50)	16 (14)	39 (35)
**Overall score**				
Hospital	7	--	4 (57)	3 (43)
Health center IV	13	4 (31)	7 (54)	2 (15)
Health center III	43	21 (49)	21 (49)	1 (2)
Health center II	48	48 (100)	--	--
Total	111	73 (66)	32 (29)	6 (5)

Of the 73 assessed government facilities, two (3%) received an overall score of >85% compared to 5 of 38 (13%) assessed private facilities (odds ratio [OR] 0.24, 95% confidence interval [CI]: 0.04–1.37, p=0.11) (Table 3). The 5 private facilities that received an overall score of >85% were all PNFPs. When compared to PNFPs alone (N=28), government facilities were less likely to achieve an overall score of >85% (OR 0.17, 95% CI: 0.03–0.98, p=0.048). Of the 73 assessed government facilities, 27 (37%) received an overall score of >70% compared to 11 of 38 (29%) assessed private facilities (OR 1.44, 95% CI: 0.62–3.36, p=0.39). Of the 20 hospital or health center IV facilities, five (25%) received an overall score of >85% compared to only one of 91 (1%) health center II or III facilities (OR 30.0, 95% CI: 3.27–274.99, *p*=0.003). Of the 20 hospital or health center IV facilities, 16 (80%) received an overall score of >70% compared to 22 of 91 (24%) health center II or III facilities (OR 12.54, 95% CI: 3.79–41.49, p<0.001) (Table 4).

**Table III:** Modified WHO Infection Prevention and Control Assessment tool scores of health facilities by ownership in Southwestern Uganda based on the Red-Amber-Green status score assessment result for each core component of the World Health Organization Infection Prevention and Control strategies

Core component	Facilities assessed, N	Number of health facilities scoring Red (<70%), N (%)	Number of health facilities scoring Amber (70–85%), N (%)	Number of facilities scoring Green (>85%), N (%)
**IPC/WASH organization**				
Government	73	12 (16)	22 (30)	39 (53)
PNFP	28	5 (18)	12 (4)	11 (39)
PFP	10	6 (60)	4 (40)	--
Subtotal	111	23 (21)	38 (34)	50 (45)
**Screening and isolation**				
Government	73	6 (8)	52 (71)	15 (20)
PNFP	28	1 (4)	22 (79)	5 (18)
PFP	10	5 (50)	4 (40)	1 (10)
Subtotal	111	12 (11)	78 (70)	21 (19)
**Hand hygiene**				
Government	73	14 (19)	57 (78)	2 (3)
PNFP	28	3 (11)	23 (82)	2 (7)
PFP	10	8 (80)	2 (20)	--
Subtotal	111	25 (22)	82 (74)	4 (4)
**Personal protective equipment**				
Government	73	33 (45)	40 (55)	0 (0)
PNFP	28	16 (57)	11 (39)	1 (4)
PFP	10	6 (60)	4 (40)	--
Subtotal	111	55 (49)	55 (49)	1 (1)
**Waste management**				
Government	73	21 (29)	50 (68)	2 (1)
PNFP	28	5 (18)	21 (75)	2 (7)
PFP	10	5 (50)	4 (40)	1 (10)
Subtotal	111	31 (28)	75 (68)	5 (5)
**Environmental cleaning**				
Government	73	10 (14)	9 (12)	54 (74)
PNFP	28	3 (11)	5 (18)	20 (71)
PFP	10	2 (10)	7 (70)	1 (10)
Subtotal	111	15 (14)	21 (19)	75 (68)
**Instrument processing**				
Government	73	37 (51)	8 (11)	28 (38)
PNFP	28	10 (36)	8 (26)	10 (36)
PFP	10	9 (90)	--	1 (10)
Subtotal	111	56 (50)	16 (14)	39 (35)
**Overall score**				
Government	73	46 (63)	25 (34)	2 (3)
PNFP	28	17 (61)	6 (21)	5 (18)
PFP	10	10 (100)	--	--
Total	111	73 (66)	32 (29)	6 (5)

**Table IV:** Univariable analysis of the association between ownership and type of health facility in Southwestern Uganda, and overall modified WHO Infection Prevention and Control Assessment tool scores for core components of the World Health Organization Infection Prevention and Control strategies

	Overall score		Odds ratio	95% Confidence interval	P value
	<70%, N	≥70 %, N			
**Ownership**					
Government	46	27	1.0	--	--
Private	27	11	1.44	0.62–3.36	0.39
PNFP	11	17	0.90	0.37–2.22	0.83
**Type of facility**					
Health center II and III	69	22	1.0	--	--
Hospital and health center IV	4	16	12.54	3.79–41.49	<0.001
	**Overall score**		**Odds ratio**	**95% Confidence interval**	**P value**

**<85 %, N**	**≥85%, N**
**Ownership**					
Government	71	2	1.0	--	--
Private	33	5	0.24	0.04–1.37	0.11
PNFP	23	5	0.17	0.03–0.98	0.048
**Type of facility**					
Health center II and III	90	1	1.0	--	--
Hospital and health center IV	15	5	30.0	3.27–274.99	0.003

## Discussion

In order to reduce the nosocomial spread of SARS-CoV-2, at least minimum IPC practices are required in health facilities that are used for the management of patients with COVID-19. To enable adherence to these practices, it is important that the facilities have the required WHO IPC core components in place as required by interim WHO guidance [6,8]. Consistent with other studies, we found that the modified WHO IPC assessment tool can be used to inform the performance of health facilities in resource limited settings with regard to the availability of the WHO IPC core components [7]. Using the modified WHO IPC assessment tool, we found that hospitals and health center IV facilities in Southwestern Uganda were more likely than health center III and health center II facilities to meet the WHO IPC core component requirements. Overall, there was no difference in the ability of government and all private facilities to meet WHO IPC core component requirements, but government facilities were less likely than PNFP facilities to meet WHO IPC core component requirements.

The WHO IPCAF framework for the assessment of IPC activities in health facilities has been validated in other settings [7]. We used a modified WHO IPC assessment tool tailored for use in Uganda where lower level health facilities are known to be deficient in some IPC components evaluated by the original IPCAF tool. IPC assessments using other assessment tools have also been conducted in similar settings [12]. Quality improvement approaches such as the Plan-Do-Study-Act cycle interventions also work in these settings and can be used to address identified gaps following IPC assessments [13]. Implementation of actions to fill identified gaps can then be put in place for a set time after which the assessment can be repeated to monitor the improvement of services at the health facilities. For example, following the IPC assessments detailed in our study, findings were shared with the staff at the facilities. Mentoring was provided regarding the identified gaps and an action plan was developed. The developed action plan included sections on the identified gaps, the activities to be put in place to have these challenges addressed, the expected outcomes, the responsible person, and the timeframe when these would be fixed. In this way, interventions can be used to improve IPC practices and decrease the transmission of SARS-CoV-2, Ebola virus, and other nosocomial pathogens.

Hospitals and health center IV facilities may have performed better than health center II and health center III facilities in IPC assessments because they have the administrative, environmental, and engineering capacity to meet the requirements for IPC strategies. Accordingly, most higher level facilities have dedicated space to allow for adequate screening and isolation of patients with suspected or confirmed COVID-19 or EVD. In addition, they often have trained IPC personnel who supervise the IPC activities in the facility. In contrast, since the provision of support and IPC supplies by the government and implementing partners to health facilities is commensurate with the projected service load, lower level health centers may lack material and human resources, including the required eight staff to constitute an IPC committee. Furthermore, most health center II facilities do not conduct surgical procedures and therefore cannot meet the requirements for instrument reprocessing, which is one of the WHO IPC core component requirements.

In Uganda, health center II and III facilities are the most accessible health facilities to patients in the community [14]. Patients with symptomatic COVID-19 are likely to initially seek treatment at a health center II or health center III facility before being referred to a designated COVID-19 treatment center, if needed. However, health center II facilities were the least prepared for all the core components for IPC strategies. For example, comprehensive PPE, which is vital for protecting health care workers and patients from contracting COVID-19 and EVD, was lacking in most of the health center II and III facilities.

The absence of a difference between government and all private facilities in their ability to meet WHO IPC core area requirements could be attributed to a difference in the abilities of PFP and PNFP facilities to put the WHO IPC core components in place. PFP facilities work on a fee-for-service basis and are not usually supported by extramural funds to meet the requirements for IPC. On their own, the PNFP facilities performed better than the government owned facilities. PNFP facilities are more likely to be supported by the government, private donors, and implementing partner organizations to have the IPC core components in place [15]. Government facilities are typically larger and with higher patient volumes than PNFPs so material and human resources may be preferentially dedicated to patient care rather than IPC practices.

Furthermore, government funding is usually released on a quarterly basis which may limit the timely application of core component requirements. It is also possible that there are differences in IPC oversight between government and private facilities, and between PFP and PNFP facilities. As determined by a locally developed Star Rating Assessment IPC tool, baseline assessments for IPC capabilities in health facilities in Tanzania found that privately owned facilities were more likely to adhere to IPC principles in comparison to government owned facilities but there was no association of facility level to adherence to IPC principles [12]. The difference in findings could be attributed in part to the use of different IPC assessment tools and the fact that our study assessed for the presence of the WHO IPC core components and not adherence to the IPC principles. Further research is required to fully understand the reasons for differences in the availability of WHO IPC core components between government, PFP, and PNFP health facilities.

Our study had limitations. The sampling process for the assessed health facilities was non-random and was therefore susceptible to selection bias. However, of the 224 health facilities in the four study districts 111 (50%) were assessed making the study findings likely generalizable to other regions of the country. We were also not able to assess the impact of the assessment and mentoring activities on the improvement of IPC activities in the visited facilities as follow up visits have not yet been completed. In addition, use of the Red, Amber, and Green assessment criteria in the modified WHO IPC assessment tool had not been used before in Uganda. However, it proved a reliable tool for assessing core component availability at lower level health facilities, which may not be easily done using the original IPCAF tool.

### Conclusion

Health facilities in resource limited settings should have the WHO IPC core components in place to enable adherence to IPC practices and to limit spread of nosocomial infections. The modified WHO IPC assessment tool can be used to assess IPC activities in health facilities in low resource settings and can guide improvements needed to meet the WHO IPC core component requirements. Using the modified WHO IPC assessment tool, we found that few facilities in Southwestern Uganda achieved >85% of IPC practices. Our findings also suggested that urgent improvement in all areas of IPC and specifically in the allocation of PPE is needed if the risk of health facility-related transmission of SARS-CoV-2 and other nosocomial infections is to be minimized.

## Credit author statement

**Richard Ssekitoleko:** conceptualization, methodology, investigation, formal analysis, project administration, writing – original draft preparation; **Emmanuel Seremba:** methodology, data curation, formal analysis, writing – original draft preparation; **Florence Waiswa:** investigation, project administration; **Doreen Nabawanuka:** methodology, investigation, project administration, writing – review and editing; **Paul Muyinda:** writing – review and editing; **Solome Okware:** methodology, investigation, project administration, writing – reviewing and editing; **Bongomin Bodo:** methodology, investigation, project administration, writing – reviewing and editing; **Yonas Tegegn Woldemariam:** supervision, methodology, project administration, writing – reviewing and editing; **Christopher C. Moore:** methodology, formal analysis, writing – original draft preparation.

## Ethics statement

As the study did not include any patient-level data, no ethics approval or patient consent was required.
